# Development of Malaria Digital Archives in Myanmar Through Official Development Assistance and Their Narrative Review

**DOI:** 10.3390/pathogens14050481

**Published:** 2025-05-14

**Authors:** Koji Kanda

**Affiliations:** Department of Social Medicine, Asahikawa Medical University, Asahikawa 078-8510, Hokkaido, Japan; kkanda@asahikawa-med.ac.jp; Tel.: +81-166-68-2402

**Keywords:** Myanmar, malaria, elimination, digital archives, Official Development Assistance, narrative review

## Abstract

The Myanmar government aims to eliminate malaria by 2030, but comprehensive summaries of its malaria control efforts are scarce. To support this goal, a literature review and long-term document preservation are essential. This study collected academic papers, master’s and doctoral theses, and policy documents on malaria control in Myanmar and its surrounding regions, published between 1950 and 2016 through Official Development Assistance. The documents were sourced from online databases, medical universities, and research institutions in Yangon. They were categorized by region and WHO-defined malaria control activity areas and archived digitally at the Ministry of Health. A total of 1107 relevant papers were identified, with 818 collected. Epidemiology-related studies accounted for 40%, followed by drug resistance, surveillance, and treatment. Recent years have seen a rise in genetic and molecular epidemiology research. Full-text analysis revealed significant malaria research in border regions, particularly near the borders of Thailand and China. This study highlights the progress and historical trends in Myanmar’s efforts to control malaria. The archive created will be a valuable resource for future policy planning and implementation efforts aimed at achieving malaria elimination.

## 1. Introduction

Malaria is a vector-borne disease that infects 249 million people and causes more than 600,000 deaths in 2022 [1]. These figures have improved significantly over the last two decades, including in Southeast Asia, where malaria cases and deaths have reduced by 76% and 77%, respectively [1]. Myanmar, one of the malaria-endemic countries, has also shown a significant decline since the early 2000s in both the number of cases and deaths. The number of infected cases decreased from 440,208 confirmed diagnosed cases in 2010 to 76,518 in 2018, and the number of deaths declined from 1261 in 2007 to 19 in 2018 [2]. The proportion of cases diagnosed as *P. falciparum* decreased from 71% in 2010 to 52% in 2018 [2]. Age-standardized malaria DALYs rates also reduced significantly from 1350 per 100,000 individuals in 2010 to 34.2 in 2019 [3]. To achieve this, Myanmar has used a larger amount of funds from the government and foreign aid for malaria control than many African countries [4]. However, while no indigenous cases have successfully reached some states and regions, infection rates still remain high around the eastern and western borders, where measures to combat the disease have not progressed sufficiently. In fact, Myanmar remains the most significant contributor to the malaria burden in the Greater Mekong Subregion (GMS), accounting for 92.4% of indigenous malaria cases and 95% of indigenous cases of *P. falciparum* [1]. Additionally, *pfKelch13* mutations associated with artemisinin partial resistance have increased prevalence in Myanmar and Thailand [1,5,6]. Therefore, the Myanmar government has set regional malaria reduction targets in its National Strategic Plan for Malaria Elimination 2021–2025, including early and effective malaria case management, universal coverage of high-risk populations with appropriate malaria prevention measures, and case-based surveillance for the elimination and prevention of re-establishment [2]. In response, effective measures such as mass administration of antimalarial drugs and malaria diagnosis and treatment support by community health workers in remote areas have begun to be put into practice [7,8,9].

An analysis of the history and the current practices of malaria elimination within the country and worldwide is essential for the government in planning and operationalizing effective malaria control measures toward its elimination. The Japanese government has been providing malaria control measures to Myanmar as part of its Official Development Assistance (ODA) since 2005 [10], despite many donor countries restricting or suspending development assistance until the democratic transition in 2011. During the restriction of ODA from many countries, NGOs and other organizations often conducted aid activities without sufficient understanding of the Myanmar government’s direction, resulting in some assistance activities that were not aligned with the government’s objectives [11]. Additionally, there has been a barrier to the systematic storage of malaria-related documents and materials in the government, as it was said that many documents were lost when the capital was relocated from Yangon to Nay Pyi Taw in 2006. Even if properly stored, most are in paper form and scattered in various locations, making them difficult to access quickly. To address the above issues, digital archives have been widely introduced in public institutions, including museums, libraries, and archives. The digital archive provides access to valuable intellectual assets anytime, anywhere, via a standard, widely accessible computer [12,13]. It will enable the secure preservation of valuable historical materials and intellectual property and facilitate the widespread dissemination and publication of the results of activities, to date, with the government taking the lead. Especially in a country like Myanmar, where the use of archives and existing materials is limited, collecting and digitizing malaria-related documents and materials in one place will not only provide a valuable source of information for developing future malaria control measures but also serve as a basis for centralizing and permanently storing records. Efficient sharing of information through the digital archive will also facilitate more effective implementation of malaria control measures, not only by the Myanmar government but also by strengthening cooperation with international organizations and NGOs. However, despite the widespread practice of developing digital archives in libraries and archives, few government agencies have undertaken such an effort specifically for materials in the health sector, including those related to malaria control and prevention.

Therefore, the purpose of the study is to develop a digital archive to systematically preserve malaria-related literature and materials necessary to achieve malaria elimination in Myanmar, to permanently provide intellectual property and historical materials to be useful for future malaria control measures developed by government agencies, and to propose a method of using the archive as a base for malaria control.

## 2. Materials and Methods

This study was part of a technical cooperation project aimed at malaria control in Myanmar, titled “The Project for Development of Malaria Elimination Model” under Japanese ODA. Initially, it was a four-year project, but it was extended for an additional two years until 2022. At the beginning of the project, one of the expected outcomes was the implementation of supportive technical assistance. This study involved several activities, such as enhancing the management capacities of the malaria elimination program within relevant official institutions and departments. Therefore, the following outputs were expected: (1) collection and analysis of academic papers and compilation of abstracts on research studies conducted in Myanmar and its bordering countries; (2) collection and compilation of policy documents and related materials on malaria control in Myanmar; (3) collection and compilation of full-text documents published in the countries where there were no indigenous malaria and/or indigenous malaria was successfully eliminated; and (4) systematic and permanent storage of malaria-related documents and materials by building a digital archive.

These outcomes correspond to the three stages of developing the digital archives: preparation, digitization, and system construction [14]. In this study, malaria-related academic literature and policy documents were collected during the preparatory stage. They were scanned and converted to digital format, and then electronically preserved in an appropriate governmental repository using commercial software.

### 2.1. Document Collection During the Preparation Stage

The collection of materials related to malaria in Myanmar included academic papers, theses and dissertation abstracts, and policy documents published from the 1950s to the end of 2016. Academic papers covered manuscripts on malaria control and prevention in Myanmar and neighboring countries. Several articles published in local journals were cataloged in the Department of Medical Research (DMR) Central Biomedical Library Database. Relevant online articles in the Myanmar Health Science Research Journal and Myanmar Medical Journal were downloaded as PDF files. However, most local articles were available in hard copy, so they were collected individually and stored in the facilities where they were located. All the collected materials were converted into PDF format. On the other hand, articles published in international journals were identified through academic databases, including the Cochrane Library, Google Scholar, J-Stage, and PubMed. In addition, three previously published abstracts found during the literature collection were also used as references [15,16,17]. The articles were downloaded from the WHO-HINARI (Health InterNetwork Access to Research Initiative) program via the university library network.

The theses and dissertations published by Myanmar universities were explored in the DMR Central Biomedical Library Database and the Union Catalog of Myanmar Health Science Libraries. Their bibliographic information was checked in advance, and the abstracts were collected in hard copy at the universities and converted to PDF format. These collected scientific documents, theses, and dissertations were briefly analyzed for their characteristics, categorizing them by publication date, study area in the country, and research topics.

Regarding the policy documents, the National Health Plan, the 5-year Malaria Strategic Plan, the Annual Public Health Statistics Report, and the Annual Hospital Statistics Report were collected and saved in electronic format. The malaria-related articles published in the government-affiliated newspaper, The New Light of Myanmar (renamed The Global New Light of Myanmar as of 1 October 2014), were collected and archived because they can serve as historical documents illustrating the progress of malaria control.

The sources of information and facilities visited for reference collection are shown in Table 1. Permissions were granted to each facility for access to materials. According to the WHO and relevant guidelines [18,19], they were analyzed thematically into 12 categories after screening to remove duplicates and irrelevant documents.

Additionally, documents from outside Myanmar, including scientific papers and other reports published in international journals and by international organizations between 2000 and 2016, were examined from 18 countries certified as malaria-free or with no indigenous cases [18]. Among them, the following seven countries were identified for document collection: Armenia, the Maldives, Morocco, Oman, Paraguay, Sri Lanka, Turkey, and Uzbekistan. Myanmar’s malaria situation, including the vector breeding environment, was similar to that in Sri Lanka, which was the Asian country recently certified as malaria-free in 2016; therefore, the collection of documents was mainly focused on Sri Lanka, and they were categorized into “Vector Control”, “Diagnosis & Treatment”, “Surveillance”, or “Other issues related to elimination”.

### 2.2. Digitization and System Construction

Digitization is the process of making hardcopy materials into electronic files. The collected documents were scanned using a Canon CanoScan LiDE220 (Canon Inc., Tokyo, Japan). The file format was PDF, with a resolution of 300 dpi. However, online documents and materials in formats other than PDF, such as Word and Excel, available in the appendix, were saved in their published formats without modification. Regarding gradation, documents printed in black and white without photographs were digitized in simple black and white. If necessary, those printed in black and white and containing photographs were digitized in 8-bit grayscale (256 shades), and color-printed documents were digitized in 24-bit full color. The image compression ratio was high. Scanning units were defined as one per facing page (A4 size). Documents that were not allowed to be removed from the libraries, such as theses and dissertations, were scanned onsite. All the scanned documents were visually checked for skewed, missing, or blurred parts before each scan. Each document was assigned a unique identification name according to a predetermined naming rule.

During system construction, the scanned materials were preserved at the project office in the government facility of vector-borne disease control. A hard disk on a designated desktop was used as the storage medium for the PDFs, and an external hard disk was used as a backup (USB 2.0 connection, 1 TB, one partition, NTFS format). The software used for the archived materials was EndNote X8 (Clarivate Plc.). Since releasing the collected documents to the general public would require extensive administrative procedures, including copyright clearance, they were made accessible internally for the time being. Additionally, a manual for browsing documents was prepared to enable users to quickly search and refer to them. The saved e-documents were also printed and filed on the bookshelves of the reference room.

## 3. Results

### 3.1. Document Collection as a Preparation Stage

**Figure 1:**
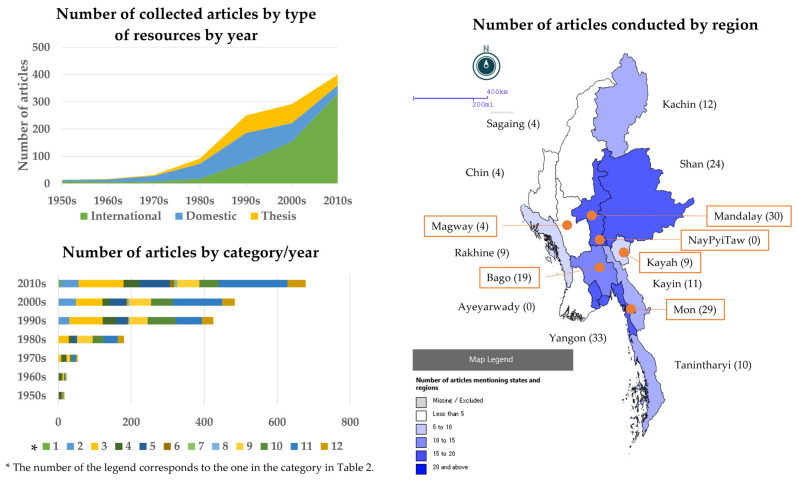
Summary of the documents related to malaria in Myanmar since the 1950s.

A total of 1107 scholarly papers from Myanmar and its bordering areas were collected, comprising 908 academic articles and 199 theses and dissertations. Among 908 articles, 818 (90.1%) were saved as full-text PDF files. Doctoral dissertations accounted for 11.1% of the 199 theses and dissertations. Regarding the field of study, 40% of the collected articles were related to epidemiology, followed by those on drug resistance, surveillance, and treatment. In recent years, the number of papers on genetic and molecular epidemiology has increased significantly. On the other hand, papers related to entomology have been published continuously since the 1950s, and research results on treatment and drug resistance have been published since relatively early times. Many documents on diagnostic techniques have been relatively recent, dating back to the 1990s, due to the widespread dissemination of rapid diagnostic tests (RDTs) (Table 2, Figure 1). When the collected materials were classified geographically in Myanmar, only 17.9% of the articles classified by title, abstract, and keywords referred to specific research areas (Figure 1). Notably, there was limited research in northwestern Myanmar, which both domestic and international organizations poorly supported. When the scope was expanded to include a full-text search, the differences in activities by region became apparent, and the publication of results in the Thai border region and the Chinese border region in the eastern part of the country stood out (Figure 1).

Regarding policy and related documents, 14 documents were accessible in the National Archives, and eight reports on malaria statistics were available. The former, in particular, covers the malaria control measures implemented from 1915 to 1975, primarily during the British colonial period and the period of transition following national independence, and includes records of activities related to the malaria eradication program led by WHO in the 1960s. Any other paper documents were in poor condition and, therefore, unacceptable for digital archiving. One to nine malaria-related articles by government newspapers were published annually since 2003, with most focusing on trends in assistance by international organizations, related conferences, and the human resource development of volunteers and others working in health centers at the project’s conclusion.

About the documents from countries certified as malaria-free or with no indigenous cases, 1821 articles were identified from online databases. Among the seven selected countries, 206 out of 534 articles were saved as full-text PDFs, which were 1 from Armenia, 2 from the Maldives, 9 from Morocco, 5 from Oman, 5 from Paraguay, 183 from Sri Lanka, 1 from Turkey, and none from Uzbekistan (Table 3). An average of 10.8 articles (Range: 4–18) were published from Sri Lanka annually, with more than half of these articles discussing vector control, diagnosis and treatment, or any mode of surveillance highlighting the latest trends in malaria outbreaks.

### 3.2. Digitization and System Construction

**Figure 2:**
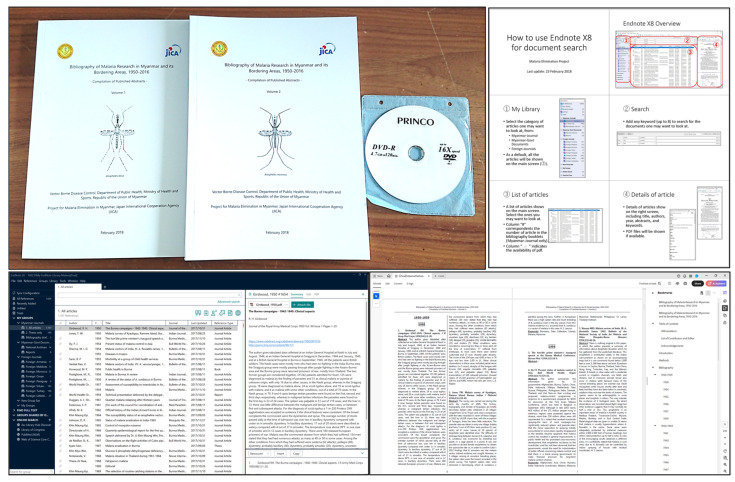
Products of digital archives.

The digital archive was established with a specified desktop in the corner of the project office’s reference room. The recorded materials, except for some electronic media content, were converted to PDF format, and text was extracted from the images using OCR technology whenever possible, enabling searching and analysis. Additionally, metadata was added to the recorded materials for easy retrieval, and guidelines were created to describe the retrieval methods (Figure 2). A list of archived documents is available in the Supplementary Materials (Table S1).

Abstracts of the collected literature were compiled into two volumes in collaboration with the Myanmar government and distributed free of charge to 59 university libraries and other relevant institutions [20,21] (Figure 2).

## 4. Discussion

The purpose of building a digital archive is to properly preserve malaria-related materials in Myanmar, enhance their accessibility, and facilitate their long-term visualization and reuse. Old documents and papers are vulnerable to physical deterioration, loss, or damage due to disasters. This study revealed that a significant number of materials, including government documents, were missing, and some of those that were collected were in poor condition. Therefore, developing a digital archive made it possible to keep them permanently and pass them on to future generations. Additionally, recent advances in information technology have significantly enhanced access to materials through digital archives, thereby facilitating their broader use and dissemination. Although further technical assistance is needed to guarantee that everyone has full access to the collected materials, the development of the malaria-related archive in a governmental facility in Myanmar was a significant achievement. And because most collected materials have been digitized, search and analysis have become more manageable. Especially in the case of papers and old documents, it is possible to collect information more efficiently by searching with specific keywords or phrases. Since the data are handled in this manner, combining it with other datasets is likely to yield new findings.

During the preparation stage, the study collected malaria documents to be securely stored in conjunction with the construction of the digital archive. As a result, 818 of the 1107 papers collected were saved as full-text PDFs, representing a significant achievement in maintaining these materials. In particular, papers related to epidemiology accounted for 40% of the total, with a notable increase in genetic and molecular epidemiology, suggesting that malaria research has shifted toward more molecular-level analysis in recent years. Advances in these areas may provide new approaches to malaria diagnosis, prevention, and treatment and represent important directions for future research. Additionally, the field analysis of the papers underscores the significance of research on drug resistance, surveillance, and treatment. Drug resistance, in particular, is one of the most significant challenges in malaria control, and ongoing research on this topic is expected to contribute to future drug development and enhanced treatment. Furthermore, the number of publications on the widespread use of rapid diagnostic tests (RDTs) has increased since the 1990s, indicating that enhanced diagnostic techniques play an essential role in malaria control. Furthermore, malaria-related articles published in government newspapers suggest that assistance, particularly from foreign aid agencies, has played a significant role in recent years. However, the absence of reports on technical cooperation suggests that technical advice and cooperation may be lacking in future assistance.

The classification of the collected literature by region indicates that less than 20% (17.9%) of the articles mention the survey region. This result clearly shows the regional bias of research studies within Myanmar. In particular, the limited number of studies conducted in the northwestern region, which is less well-supported, reflects the regional inequality. In contrast, research results on the Thai and Chinese borders were remarkable, showing the strong influence of external support. The differences in research by region reflect regional disparities in resource allocation and support in malaria control, suggesting that future research and support activities should be balanced to meet the needs of each area. The number of policy documents was relatively small because Myanmar had a long history of malaria control without formal documentation during the long, undemocratic regime.

During digitization and system construction, some of the collected materials were in a poor state of preservation, including items that could not be preserved as digital archives. This highlights an essential issue in the conservation and management of materials. Digital archives are a necessary means of preserving materials for the long term and making them widely accessible; technical innovations are needed to digitize and preserve materials that are poorly maintained. Advances in techniques for restoring and digitizing deteriorating materials will increase the likelihood that these valuable materials will be passed on to future generations. In this study, the basic design of the digital archive has been completed. To operate the system permanently and update the stored information regularly, sufficient funds and resources will be required after the project is completed. Long-term support must be secured through collaboration with government agencies, academic institutions, and private companies. The system has been built using open software and other cost-saving methods. Still, it will be necessary to update and improve the system as new materials are added and technology advances. Utilizing AI to automatically classify and tag materials will enhance the management and search accuracy of materials, thereby providing a more user-friendly environment for users. There are also attempts to visualize materials in new ways using virtual reality (VR) and augmented reality (AR) technology. Human resource development is also necessary. Therefore, it will be required to ensure a sustainable management system.

Limitations of this study include legal and ethical considerations regarding the widespread availability of digital archives to the public. In particular, copyrights of digitized materials need to be carefully addressed. If the materials are to be made widely available to the public, the copyrights to the archived materials must be verified, and permission must be obtained from the copyright holders. Although it is rare for this study to include personal or privacy-related content, regulations and considerations regarding handling such personal information are necessary. For this reason, this study was limited to viewing within the facility only, and access to the outside world was restricted. On the other hand, a separate collection of abstracts was compiled for the stored academic papers, creating an environment that facilitates easy searches within the archive. It is also crucial to support users effectively and accurately when using digital archives. For this reason, guidelines were created to establish an environment that informs users of the archives’ benefits and how to use it effectively.

This study aimed to collect and digitally save materials essential for understanding the historical context of malaria control in Myanmar, as well as the current state of research and policy, and to provide recommendations for early malaria elimination. Collecting and archiving scientific papers demonstrated advances in epidemiology, genetic epidemiology, and diagnostic techniques, raising expectations for future research. At the same time, understanding research trends by region could increase awareness of regional inequalities and demand more effective support. Furthermore, the collection of past policy documents has provided lessons learned for future malaria control, reaffirming the importance of digital archives. To effectively utilize these documents in the future, along with technical assistance, flexible support tailored to the needs of each region is needed.

## Figures and Tables

**Table 1:** Resources for digital archive references.

**Libraries and Institutions Visited in Myanmar**	**URL (accessed on 15 November 2016)**
Department of Medical Research (DMR) Central Biomedical Library	http://www.dmrlibrary.gov.mm/
Myanmar Medical Association Library	https://www.mmacentral.org/library/
National Archives of Myanmar	https://www.nam.gov.mm/Archives.aspx
National Library of Myanmar	http://www.nlm.gov.mm/
Universities Central Library	https://www.uclmyanmar.org/
University of Medicine (1)	http://um1yangon.edu.mm/en/
University of Medicine (2)	https://um2ygn.edu.mm/
University of Yangon	https://www.uy.edu.mm/
**Major local journals**	**Issue Year**
Department of Medical Research Annual Report	1963–
Department of Medical Research Bulletin	1986–
E-Bulletin of Preventive and Social Medicine Society	2014–
Journal of Burma Studies	1957–
Journal of Myanmar Military Medicine	1992– (Occasional publication)
Medical Education Unit Bulletin, UM Mandalay	2001–
Myanmar Health Science Research Journal	1989–
Myanmar Journal of Current Medical Practice	1996–
Myanmar Medical Journal (Burma Medical Journal)	1953–
Myanmar Nursing and Midwifery Journal	2006–
Myanmar Pediatric Journal	Around 2002–
Pediatric Bulletin	Around 1984–
Union of Burma Journal of Life Sciences	1968–1972
University of Nursing Mandalay Nursing Journal	2014–
**Local and international online database**	**URL (accessed on 15 November 2016)**
DMR Central Biomedical Library database (for only domestic articles, theses, and dissertations)	http://www.dmrlibrary.gov.mm/
Online Burma/Myanmar Library -The Global New Light of Myanmar	http://www.burmalibrary.org/show.php?cat=1450
Union Catalog of Myanmar Health Science Libraries (Only theses and dissertations)	http://www.healthscienceucat.com.mm/
UNICEF office	https://www.unicef.org/myanmar/
WHO Archives	http://www.who.int/archives/en/
WHO Myanmar	http://www.searo.who.int/myanmar/en/
International search engines (PubMed, Google Scholar, Cochrane Library, Web of Science, J-Stage)	Refer to each engine.

**Table 2:** Number of articles by detailed research category (N = 1107, Multiple responses).

Category	Number	%
Community case management (iCCM)	7	0.6
2. Diagnostic testing (RDT, microscopy, nucleic acid amplification-based diagnostics)	127	11.5
3. Drug resistance and response (drug efficacy, pharmacokinetics, clinical trial, traditional medicine)	325	29.4
4. Entomology and vector control (LLIN, IRS, entomological surveillance, insecticide resistance)	136	12.3
5. High-risk groups (pregnant women, infants, children under five years of age, patients with HIV/AIDS, non-immune migrants, mobile population, travelers)	177	16.0
6. Malaria elimination (malaria eradication program, sustained political commitment, adequate resourcing, effective partnerships, prevention of reintroduction)	23	2.1
7. Malaria vaccine development (RTS, S/ASO1, Pvs48/45)	9	0.8
8. Preventive therapies (intermittent preventive treatment, seasonal malaria chemoprevention)	8	0.7
9. Surveillance (epidemiological, immunological, serological)	226	20.4
10. Treatment (ACT, safe and effective quality antimalarial medicines, withdrawal of oral artemisinin-based monotherapies, procurement, and supply chain management)	227	20.5
11. Epidemiology (behavioral, genetic, molecular, serological, social)	450	40.7
12. Others	145	13.1

**Table 3:** Summary of collected documents outside Myanmar.

Country/Category	Articles After Screening	Full-Text PDF
Armenia	17	1
Maldives	73	2
Morocco	23	9
Oman	7	5
Paraguay	6	5
Sri Lanka	183	183
1. Vector Control (LLIN, ITN, Entomology)		24
2. Diagnosis and Treatment		20
3. Surveillance (Epidemiological, Serological, Entomological)		57
4. Other issues related to elimination		82
Turkey	198	1
Uzbekistan	27	0

## Data Availability

The original contributions presented in this study are included in the article/Supplementary Materials. Further inquiries can be directed to the corresponding author.

## Supplementary Materials

The following supporting information can be downloaded at: https://www.mdpi.com/article/10.3390/pathogens14050481/s1, Table S1: A complete list of collected malaria-related documents in Myanmar and adjacent countries with subject index.
